# STMN1 Promotes Tumor Metastasis in Non-small Cell Lung Cancer Through Microtubule-dependent And Nonmicrotubule-dependent Pathways

**DOI:** 10.7150/ijbs.84738

**Published:** 2024-02-07

**Authors:** Lizhong Zeng, Xin Lyu, Jingyan Yuan, Yang Chen, Haimei Wen, Lei Zhang, Jie Shi, Boxuan Liu, Wei Li, Shuanying Yang

**Affiliations:** 1 Department of Respiratory and Critical Care Medicine, Second Affiliated Hospital, Xi'an Jiaotong University, No. 157, Xiwu Road, Xincheng District, Xi'an 710004, Shaanxi, P.R. China.; 2 Department of Pharmacy, Shaanxi Provincial Hospital of Chinese Medicine, Xi'an, China, Xi'an 710061, Shaanxi, P.R. China.

**Keywords:** STMN1, HMGA1, p38MAPK/STAT1, Epithelial-mesenchymal transition, Non-small cell lung cancer

## Abstract

The relationship between STMN1 and cancer metastasis is controversial. The purpose of this study was to explore the role and mechanism of STMN1 in NSCLC metastasis. In this study, we reported that STMN1 was highly expressed in NSCLC tissues and associated with poor prognosis. Both *in vivo* and *in vitro* functional assays confirmed that STMN1 promoted NSCLC metastasis. Further studies confirmed that STMN1 promoted cell migration by regulating microtubule stability. The results of Co-IP and LC‒MS/MS illustrated that STMN1 interacts with HMGA1. HMGA1 decreases microtubule stability by regulating the phosphorylation level of STMN1 at Ser16 and Ser38 after interacting with STMN1. This result suggested that STMN1 could be activated by HMGA1 to further promote NSCLC metastasis. Meanwhile, it has been found that STMN1 could promote cell migration by activating the p38MAPK/STAT1 signaling pathway, which is not dependent on microtubule stability. However, activating p38MAPK can decrease microtubule stability by promoting the dephosphorylation of STMN1 at ser16. A positive feedback loop was formed between STMN1 and p38MAPK to synergistically promote cell migration. In summary, our study demonstrated that STMN1 could promote NSCLC metastasis through microtubule-dependent and nonmicrotubule-dependent mechanisms. STMN1 has the potential to be a therapeutic target to inhibit metastasis.

## Background

Lung cancer is the leading cause of cancer death, accounting for 18% of all cancer-related deaths^1^. Lung cancer can be divided into small cell lung cancer and non-small cell lung cancer (NSCLC), of which the latter accounts for more than 85%^2^, ^3^. In recent years, with the popularization of low-dose spiral computed tomography and the development of targeted therapy and immunotherapy, the five-year survival rate of NSCLC patients has improved, but it is still not ideal^4^. Metastasis is one of the main factors contributing to poor prognosis in NSCLC patients^5^, and epithelial-mesenchymal transition (EMT) is considered the key process of cancer metastasis^6^, ^7^. Exploring potential therapeutic targets for targeted inhibition of metastasis is critical to improving lung cancer prognosis.

STMN1 is a microtubule destabilizing protein widely distributed in the cytoplasm^8^. STMN1 has four phosphorylation sites, Ser16, Ser25, Ser38 and Ser63, and its dephosphorylated state is the active form. STMN1 can promote microtubule depolymerization when it is activated by dephosphorylation, whereas it can stabilize microtubules when phosphorylated^9^, ^10^. STMN1 is highly expressed in most tumors, such as breast cancer^11^, gastric cancer^12^, and hepatocellular carcinoma^13^. With the in-depth study of STMN1, it was found that it is closely related to biological processes such as proliferation^14^ and drug resistance^15^. Some retrospective clinical studies have found that the expression of STMN1 is positively correlated with lymph node metastasis and clinical stage in most tumors^16^, ^17^. Studies have reported that some genes can promote tumor metastasis by inhibiting the phosphorylation level of STMN1^18^, ^19^. However, there is also a different view on the role of STMN1 in tumor metastasis; for example, a study showed that STMN1 was negatively related to EMT^20^. In this study, we verify the role of STMN1 in NSCLC metastasis and explore the possible mechanisms by which STMN1 promotes NSCLC metastasis.

Microtubule dynamics play an extremely important role in various biological processes. The relationship between microtubule stability and cell migration is inseparable^21^. Paclitaxel^22^ is a microtubule stabilizer and is widely used in antitumor therapy, while colchicine^23^ is a tubulin inhibitor that can inhibit microtubule polymerization and decrease microtubule stability. The acetylation level of α-tubulin (Ace-α-tubulin) is considered to be an evaluation index of microtubule stability, and its expression level decreases when microtubule stability decreases^24^-^26^. Considering that STMN1 is a microtubule-associated protein, we used paclitaxel and colchicine in this study to systematically verify that STMN1 can promote NSCLC metastasis by regulating microtubule stability.

To better understand STMN1, coimmunoprecipitation (Co-IP) and LC‒MS/MS were further used to identify the molecules that might interact with STMN1. HMGA1 is a potential protein that interacts with STMN1 and was reported to be significantly associated with tumor metastasis^27^, ^28^. In this study, we further explored the relationship among STMN1, HMGA1, microtubule stability and tumor metastasis.

In our previous study^29^, it was found that the expression level of STMN1 was positively correlated with the phosphorylation level of p38MAPK. A large number of studies have shown that the activation of p38MAPK could promote the occurrence of tumor metastasis 30-32. STAT1 is a downstream molecule of p38MAPK that can be phosphorylated and activated by p38MAPK at the S727 site^33^. SB203580^34^ is a small molecule inhibitor that can inhibit the catalytic activity of p38MAPK toward downstream molecules, and fludarabine^35^ is a purine analog that can inhibit STAT1 expression and activation. Here, we evaluated the role of p38MAPK/STAT1 in STMN1 promotion of NSCLC metastasis and explored the relationship between p38MAPK/STAT1 and microtubule stability.

In this study, we focused on verifying the role of STMN1 in NSCLC metastasis and exploring the possible mechanisms by which STMN1 promotes NSCLC metastasis, which provides a theoretical basis for STMN1 as a therapeutic target for inhibiting metastasis.

## Materials and methods

### Clinical samples and bioinformatics analysis

In our study, 21 paired LUAD and 13 paired LUSC tumor and matched adjacent nontumor tissues were obtained from the Second Affiliated Hospital of Xi'an Jiaotong University (Xi'an, China) from January 2016 to December 2019. All participating patients in our study signed written informed consent forms, and our study was approved by the Clinical Research Ethics Committee of the Second Affiliated Hospital of Xi'an Jiaotong University (No. 2016036). The GEPIA database was used to evaluate mRNA expression^36^, the CPTAC database was used to evaluate protein expression 37, and the KM plotter database was used to evaluate the prognostic value of target genes^38^.

### Cell culture

The human NSCLC cell lines PC-9 and NCI-H1299 were purchased from the Cell Bank of the Chinese Academy of Science (China). Both cell lines were cultured in RPMI-1640 (Gibco) medium with 10% fetal bovine serum (Biological Industries, Israel). The cell incubator (Thermo) maintained a humid atmosphere containing 5% CO2 at 37 ℃.

### CRISPR/Cas9 and lentiviral transfection

The sgRNAs of STMN1 were designed according to the sequence of STMN1. The sgRNA lentiviral vector carries GFP, and the Cas9 lentiviral vector carries puromycin resistance, both of which are synthesized by GeneChem (GeneChem Biotechnology Co., Ltd., Shanghai, China). The lentiviral overexpression vector carried puromycin resistance and GFP and was also synthesized by GeneChem Biotechnology Co., Ltd. (Shanghai, China). All lentiviruses were transfected according to the manufacturer's instructions. The sequences of the sgRNAs are listed in supplementary file: Table S1.

### Xenograft studies

The animal experiments in our study were approved by the Laboratory Animal Center of Xi'an Jiaotong University. We purchased 5-week-old female nude mice from Beijing Vital River Laboratory Animal Technology Co., Ltd. (Beijing, China) and kept them in specific pathogen-free (SPF) rooms of the Animal Center of Xi'an Jiaotong University. All nude mice were randomly divided into groups and injected with targeted cells through the caudal vein. Six weeks later, the mice were sacrificed after anesthesia, and the formation of metastatic tumors in the lungs of nude mice was observed with an *in vivo* small animal imaging system.

### Immunocytochemistry

Targeted cells were seeded in 96-well plates at an appropriate density. After culturing for 48 h, the cells were fixed with formaldehyde, permeated with Triton-100, and blocked with 5% milk for 1 h. The primary antibody was incubated overnight at 4 °C, and the secondary antibody was incubated for 1 h at room temperature. Then, the cells were stained with DPIA. Finally, images were collected using a fluorescence microscope (Nikon, Tokyo, Japan). All primary antibodies were purchased from CST Biotechnology and diluted 1:300. The secondary antibody was purchased from Immunoway Biotechnology and diluted 1:500.

### Inhibitors

The inhibitors of p38MAPK SB203580 and BMS-582949, the inhibitor of STAT1 fludarabine, the calmodulin kinase inhibitor KN-62, the microtubule stabilizer paclitaxel, and the microtubule stability inhibitor colchicine were all purchased from Tsbiochem Biotechnology (Shanghai, China). All inhibitors were dissolved in dimethyl sulfoxide (DMSO) and diluted to the final concentration in complete medium. In our study, the final concentration of SB203580 was 0.5 µM, BMS-582949 was 10 nM, fludarabine was 50 µM, KN-62 was 1 µM, paclitaxel was 10 nM and colchicine was 3 nM.

### Real-time quantitative polymerase chain reaction (RT‒qPCR)

Total RNA from NSCLC cells and tissues was extracted using FAST200 (Pioneer, China) and FAST1000 (Pioneer, China) kits, respectively. A PrimerScript™ RT (Takara, Japan) kit was used to reverse transcribe RNA into complementary DNA (cDNA), and cDNA was analyzed by RT‒qPCR using a TB Green® Premix Ex Taq™ II (TaKaRa, Japan) kit. All primers used in this study were synthesized by Sangon Biotech Co., Ltd. (Shanghai, China), and the primer sequences are listed in the supplementary file: Table S2. The 2^‑ΔΔCq^ method^39^ was used to analyze the relative expression levels of target genes, and GAPDH was used as an internal reference.

### Wound healing assay

Cells were seeded in 6-well plates, and a straight line was scratched with a 100 µL tip when cell confluence reached 90%. The exfoliated cells were washed with PBS and cultured with medium containing 1% FBS. The wound area was recorded under a microscope (Nikon, Japan) at 0 h and 48 h.

### Transwell migration assay

Cells were diluted with serum-free medium, and the cell concentration was adjusted to the appropriate density. Two hundred microliters of diluted cells were seeded into the upper chamber, while 600 µL of complete medium was added to the lower chamber. After 24 h, the cells were fixed with 4% paraformaldehyde for 20 min and stained with crystal violet for 20 min. The cells in the upper chamber were gently wiped, and the cells in the lower chamber were photographed and counted under a microscope (Nikon, Japan).

### Western blotting

Total protein from cells was extracted using radioimmunoprecipitation assay (RIPA) buffer (Beyotime) containing protease and phosphatase inhibitors (Roche). The proteins were separated in SDS-polyacrylamide gels and electrotransferred to polyvinylidene difluoride (PVDF) membranes. The membranes were blocked in 5% milk for 2 h at room temperature. The primary antibody was incubated overnight at 4 °C, and the secondary antibody was incubated for 1 h at room temperature. Primary antibodies against STMN1 and HMGA1 were purchased from Abcam Biotechnology, and other antibodies were purchased from CST Biotechnology. In this study, all primary antibodies except for GAPDH were diluted 1:1000, GAPDH was diluted 1:5000, and the secondary antibody was diluted 1:10000. GAPDH was used as the internal reference. Signals were detected using an HRP Chemiluminescent Kit (Millipore) and CCD camera image system (Biotanon, Shanghai).

### Coimmunoprecipitation (Co-IP) and liquid chromatography‒mass spectrometry/mass spectrometry (LC‒MS/MS)

Total protein from STMN1-overexpressing NCI-H1299 cells was extracted using IP lysis (Beyotime) buffer containing protease and phosphatase inhibitors (Roche). The primary antibodies were incubated with the magnetic beads (Millipore) for 30 min at room temperature. Next, the antibody beads were incubated with the IP protein overnight at 4 °C. In this way, STMN1 purified protein was obtained. The purified proteins were separated in SDS-polyacrylamide gels, and the PAGEs were sent to LC-Bio (Hangzhou, China) for LC‒MS/MS identification. The identified proteins that may interact with STMN1 were further verified by western blotting.

### Small interfering RNA (siRNA) and plasmid transfection

The siRNAs targeting HMGA1 and the negative control siRNA were synthesized by GenePharma (Shanghai, China). The full-length human HMGA1 cDNAs and corresponding negative control cDNA were synthesized and cloned and inserted into the expression vector PEX-3 plasmid by GenePharma (Shanghai, China). In this study, plasmids and siRNAs were transfected into targeted cells using Lipofectamine 2000 (Invitrogen, MA, USA), and the sequences of siRNAs are listed in the supplementary file: Table S3.

### Statistical analysis

All experimental data are presented as the means ± SDs. Student's t test was used to analyze significant differences between two groups. All data were statistically analyzed using GraphPad Prism 9. *P*<0.05 was considered statistically significant.

## Results

### STMN1 is highly expressed in NSCLC tissues and associated with poor prognosis

**Figure 1:**
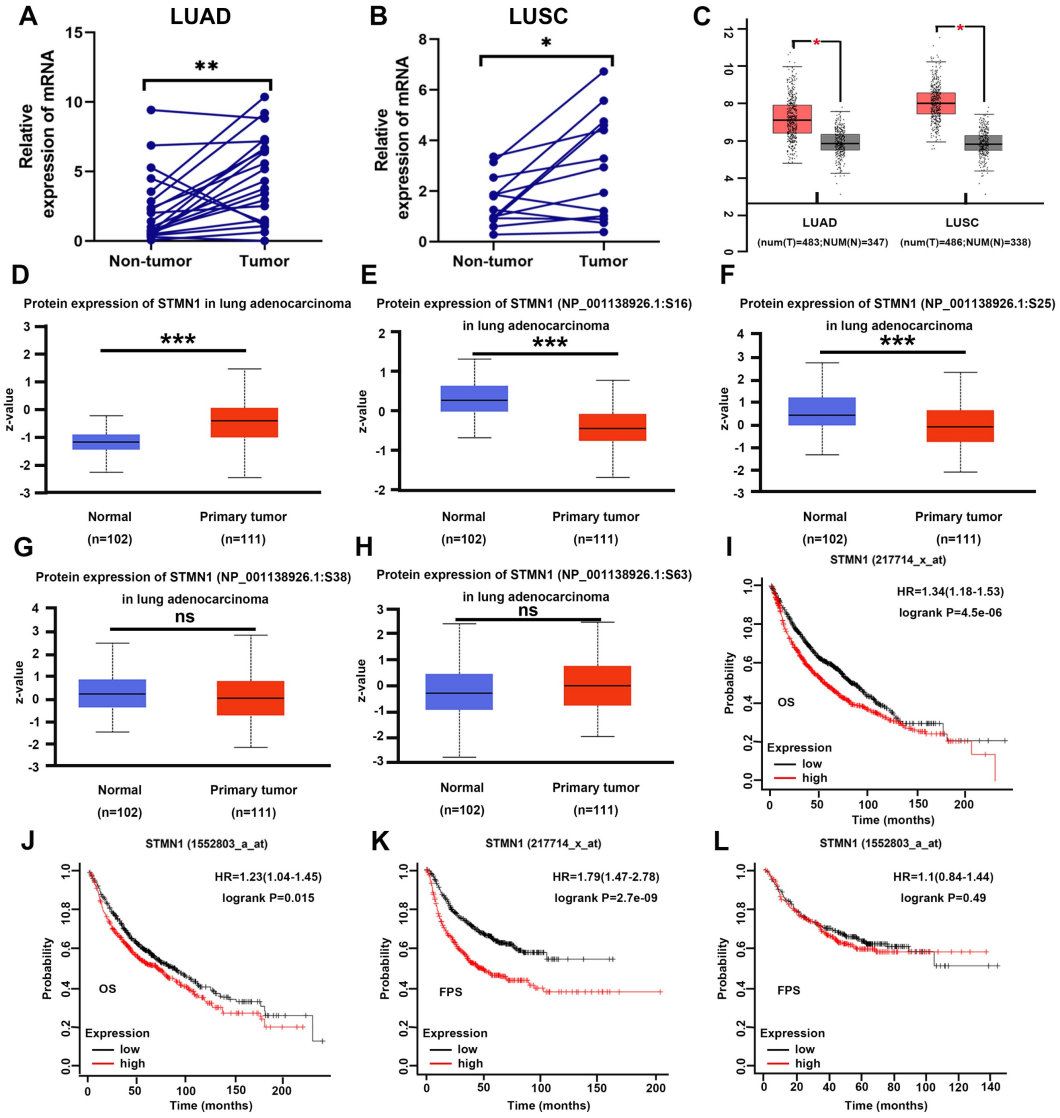
** STMN1 was highly expressed in NSCLC tissues and was related to poor prognosis. (A)** Expression of *STMN1* mRNA in 21 paired LUAD tumor tissues and matched adjacent nontumor tissues. **(B)** Expression of *STMN1* mRNA in 13 paired LUSC tumor tissues and matched adjacent nontumor tissues. **(C)** Expression of *STMN1* mRNA in LUAD and LUSC tumor and nontumor tissues in the GEPIA database. **(D)** Expression of STMN1 total protein in LUAD tumor and normal tissues in the CTPAC database. **(E-H)** Expression of STMN1 phosphorylation protein in LUAD tumor and normal tissues in the CTPAC database. **(I and J)** The relationship between *STMN1* mRNA and the overall survival of NSCLC patients in the KM plotter database. **(K and l)** The relationship between STMN1 mRNA and the free progression survival of NSCLC patients in the KM plotter database. Abbreviations: LUAD: lung adenocarcinoma; LUSC: lung squamous cell carcinoma; NSCLC: non-small cell lung cancer; GEPIA: gene expression profiling interactive analysis; CTPAC: clinical proteomic tumor analysis consortium; OS: overall survival; FPS: free progression survival; KM: Kaplan Meier. *P<0.05, **P<0.01, ***P<0.001.

In our previous studies^29^, we found that STMN1 was highly expressed in LUAD tissues and associated with lymph node metastasis by tissue microarray combined with immunohistochemical staining. In this study, *STMN1* mRNA was also found to be significantly more highly expressed in 21 LUAD and 13 LUSC tumor tissues than in matched adjacent nontumor tissues (Fig. 1A and 1B). In the GEPIA database, the results illustrated that *STMN1* was highly expressed in LUAD and LUSC tumor tissues compared with nontumor tissues (Fig. 1C). In the CPTAC database, the results showed that STMN1 total protein in LUAD tumor tissues was significantly higher than that in nontumor tissues (Fig. 1D). In addition, the results from the CPTAC database showed that the phosphorylation levels of STMN1 at ser16 and ser25 sites were significantly higher in LUAD nontumor tissues (Fig. 1E and 1F), but the expression of STMN1 phosphorylated at Ser38 and Ser63 was not significantly different (Fig. 1G and 1H). These results indicated that STMN1 was highly expressed at both the mRNA and protein levels in NSCLC tumor tissues, and activated STMN1 was also highly expressed in NSCLC tissues.

To further clarify the role of STMN1 in NSCLC, we evaluated the prognostic value of STMN1 in the KM plotter database. Probes 217714_x_at and 1552803_a_at both suggested that the patients in the *STMN1* higher expression group had worse overall survival (OS) (Fig. 1I and IJ). In the two probes, the median OS was 54.3 and 70 months in the higher expression groups and 81.2 and 87.7 months in the lower expression groups, respectively. In addition, probe 217714_x_at showed that the patients in the *STMN1* higher expression group tended to have a worse free progression survival (FPS) (Fig. 1K), and the medial FPS was 45.08 months in the higher expression group and 164 months in the lower expression group. Probe 1552803_a_at showed no significant relationship between FPS and STMN1, but the medial FPS was 25.5 months in the higher expression group and 26.3 months in the lower expression group (Fig. 1L). The above results indicated that higher STMN1 was associated with poor prognosis.

### STMN1 promotes NSCLC metastasis *in vitro* and *in vivo*

**Figure 2:**
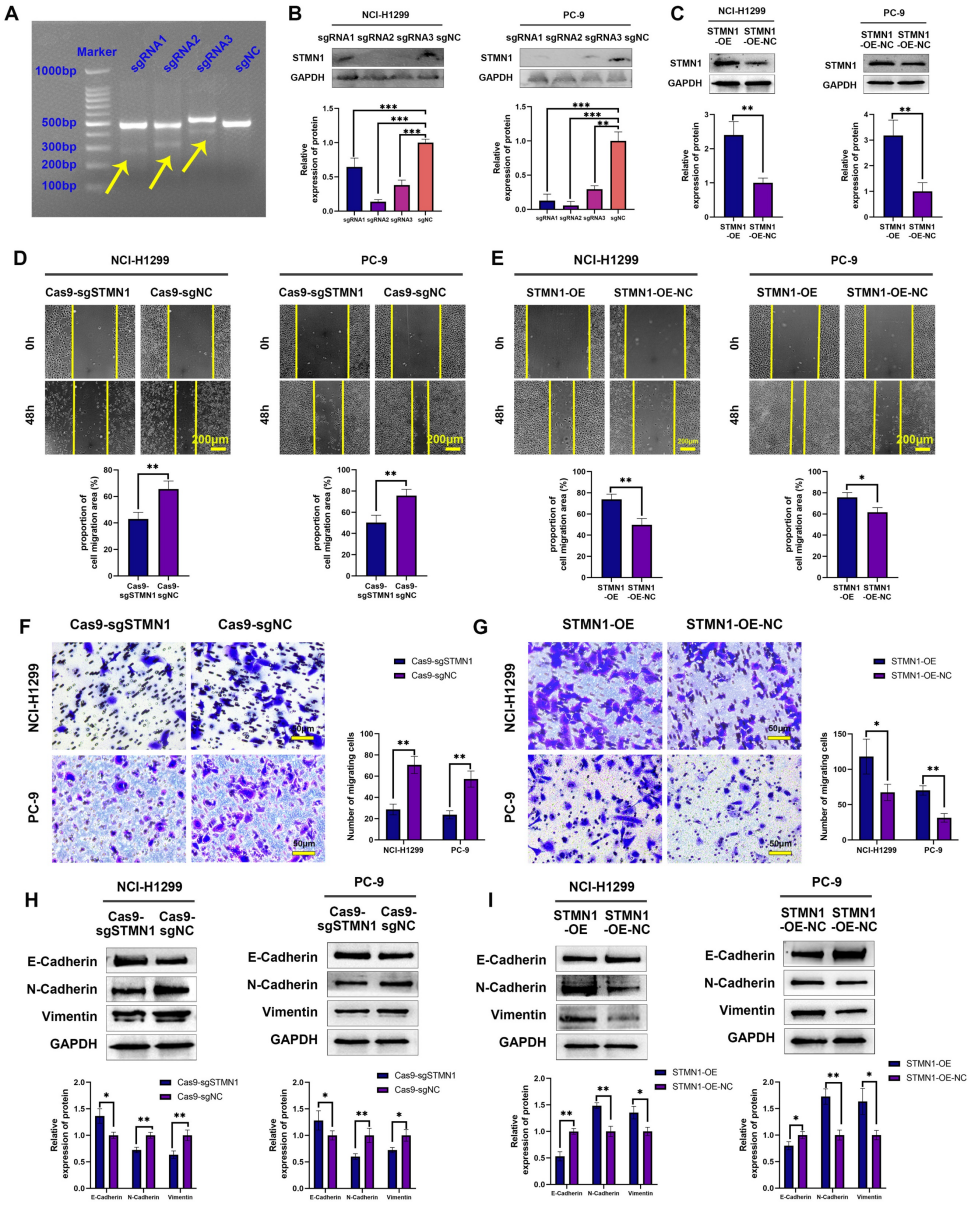
** STMN1 promotes NSCLC metastasis *in vitro*. (A)** The STMN1 knockdown efficiency in NCI-H1299 cells was evaluated by mismatched enzyme experiment. **(B)** STMN1 knockdown efficiency in NCI-H1299 and PC-9 cells were tested by western blotting. Gray analysis of STMN1 protein expression is shown in lower panel. **(C)** STMN1 overexpression efficiency in NCI-H1299 and PC-9 cells were tested by western blotting. Gray analysis of STMN1 protein expression is shown in lower panel. **(D)** In NCI-H1299 and PC-9 cells, wound healing assay in STMN1 knockdown and negative control groups. The closure rates of all wounds at 48 h were analyzed and displayed in the lower panel. **(E)** In NCI-H1299 and PC-9 cells, wound healing assay in STMN1 overexpression and negative control groups. The closure rates of all wounds at 48 h were analyzed and displayed in the lower panel. **(F)** In NCI-H1299 and PC-9 cells, transwell migration assay in STMN1 knockdown and negative control groups. The relative migrating cell numbers are analyzed in the right panel.** (G)** In NCI-H1299 and PC-9 cells, transwell migration assay in STMN1 overexpression and negative control groups. The relative migrating cell numbers are analyzed in the right panel.** (H)** In NCI-H1299 and PC-9 cells, the expression of EMT markers in STMN1 knockdown and negative control groups were detected by westering blotting. Gray analysis of EMT markers expression is shown in lower panel. **(I)** In NCI-H1299 and PC-9 cells, the expression of EMT markers in STMN1 overexpression and negative control groups were detected by westering blotting. Gray analysis of EMT markers expression is shown in lower panel. Abbreviations: NSCLC: non-small cell lung cancer; EMT: epithelial-mesenchymal transition. *P<0.05, **P<0.01, ***P<0.001.

To further explore the role of STMN1 in NSCLC, STMN1 knockdown and overexpression cell lines were constructed by transfecting CRISPR/Cas9 and overexpression lentivirus in PC-9 and NCI-H1299 cells. The results of the mismatched enzyme experiment indicated that all three sgRNAs successfully knocked down STMN1 (Fig. 2A). The results of RT‒qPCR and western blotting further indicated that three sgRNAs successfully inhibited the expression of STMN1 at the mRNA (Fig. S1) and protein (Fig. 2B) levels. The knockout efficiency of sgRNA2 was the highest, so sgRNA2 was selected for further experiments. STMN1 was also successfully overexpressed at the mRNA (Fig. S1) and protein (Fig. 2C) levels by transfecting an overexpression lentivirus. In this way, STMN1 knockdown and STMN-overexpressing cell lines were successfully acquired.

**Figure 3:**
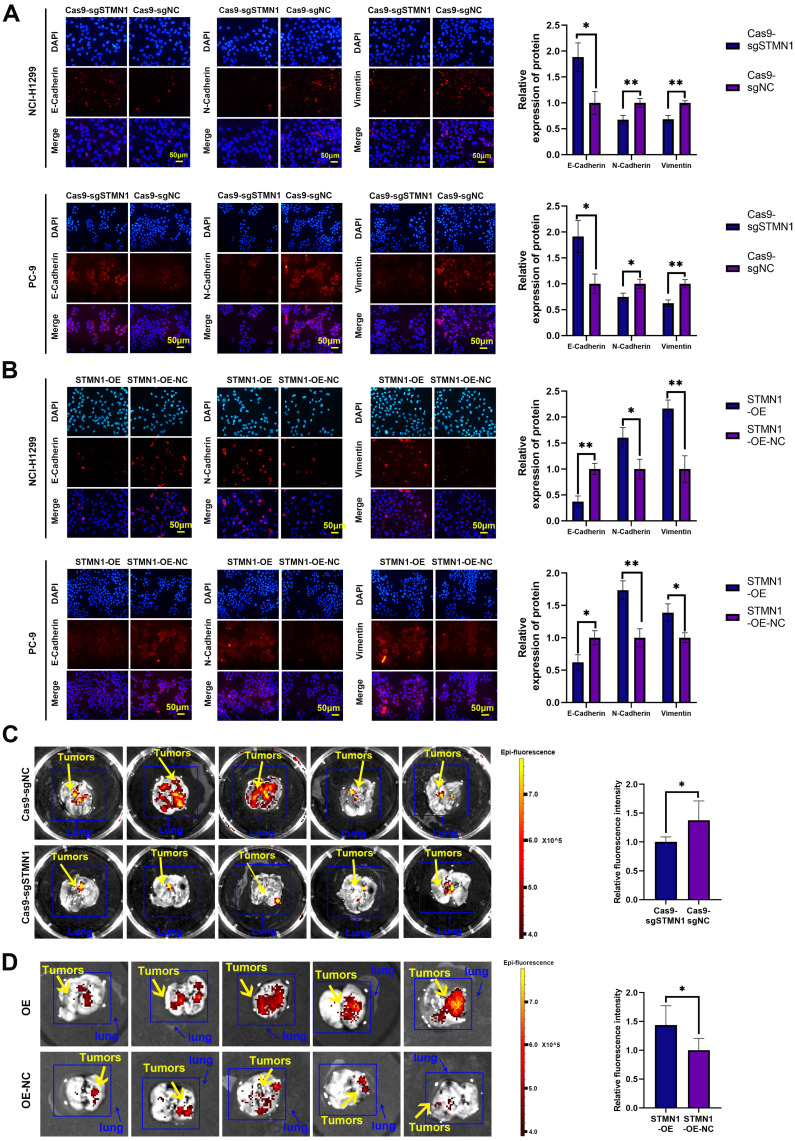
** STMN1 promotes NSCLC metastasis *in vitro* and* in vivo*. (A)** In NCI-H1299 and PC-9 cells, the expression of EMT markers in STMN1 knockdown and negative control groups were detected by immunocytochemistry. Relative fluorescence quantitative analysis is shown in right panel. **(B)** In NCI-H1299 and PC-9 cells, the expression of EMT markers in STMN1 overexpression and negative control groups were detected by immunocytochemistry. Relative fluorescence quantitative analysis is shown in right panel. **(C)** The xenograft model mice were constructed by injecting the same number of STMN1 stable knockdown and corresponding negative control cells (NCI-H1299), and the isolated lungs from xenograft model mice were captured with *in vivo* small animal imaging system. Relative fluorescence intensity analysis is shown in right panel.** (D)** The xenograft model mice were constructed by injecting the same number of STMN1 stable overexpression and corresponding negative control cells (NCI-H1299), and the isolated lungs from xenograft model mice were captured with in vivo small animal imaging system. Relative fluorescence intensity analysis is shown in right panel. Abbreviations: NSCLC: non-small cell lung cancer; EMT: epithelial mesenchymal transition. *P<0.05, **P<0.01.

*In vitro*, wound healing assays, transwell migration assays and the expression of EMT markers were used to evaluate cell migration. The results of wound healing assays showed that knockdown of STMN1 significantly inhibited the scratch healing ability (Fig. 2D), but overexpression of STMN1 enhanced the scratch healing ability (Fig. 2E). The results of transwell migration assays showed that knockdown of STMN1 significantly reduced the number of migrating cells (Fig. 2F), while overexpression of STMN1 increased the number of migrating cells (Fig. 2G). The results of western blotting showed that knockdown of STMN1 significantly increased the expression of the epithelial marker E-cadherin, and decreased the expression of the mesenchymal markers N-cadherin and vimentin (Fig. 2H), while overexpression of STMN1 inhibited the expression of the epithelial marker E-cadherin, and increased the expression of the mesenchymal markers N-cadherin and vimentin (Fig. 2I). As shown in Fig. 3A and 3B, the results of immunocytochemistry also indicated that knockdown of STMN1 promoted the expression of E-Cadherin and inhibited the expression of N-Cadherin and Vimentin, while overexpression of STMN1 has the opposite effect. These results indicated that STMN1 promoted NSCLC cell migration *in vitro*.

*In vivo*, STMN1 stable knockdown and STMN1-overexpressing cells (NCI-H1299) and corresponding negative control cells were injected into nude mice through the tail vein to observe the formation of metastatic tumors. The *in vivo* small animal imaging system was used to capture the formation of lung metastases in mice, and the fluorescence intensity and area were used to evaluate the relative number of metastases. The results showed that the relative number of lung metastases in the STMN1 knockdown group was significantly less than that in the negative control group (Fig. 3C), while the relative number of lung metastases in the STMN1 overexpression group was significantly more than that in the negative control group (Fig. 3D). These results indicated that STMN1 promoted NSCLC metastasis *in vivo*.

### STMN1 promotes NSCLC metastasis by regulating microtubule stability

**Figure 4:**
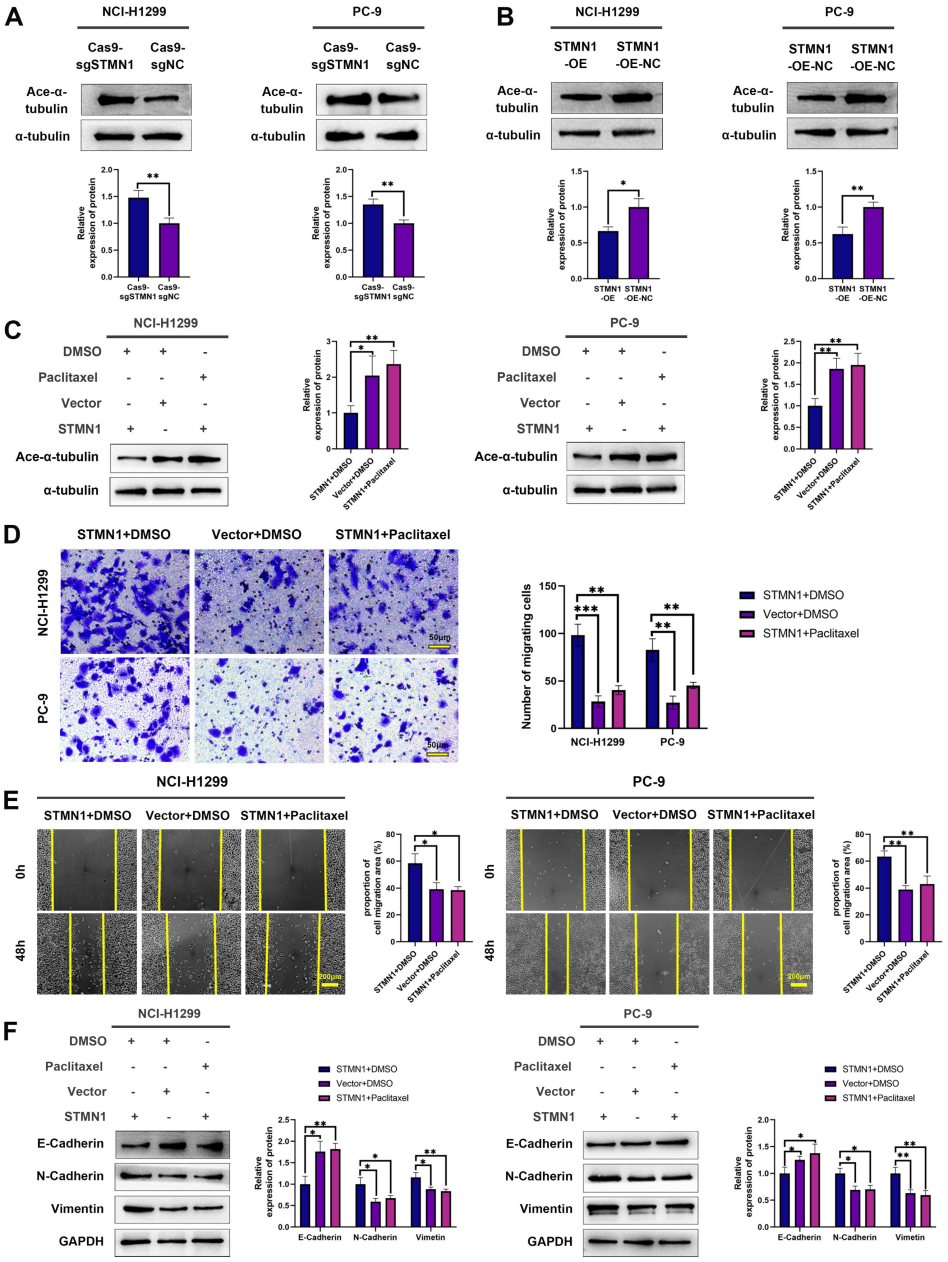
** STMN1 promotes NSCLC metastasis by regulating microtubule stability. (A)** In NCI-H1299 and PC-9 cells, the acetylation levels of α-tubulin in STMN1 knockdown and negative control groups were detected by westering blotting. Gray analysis of the acetylation levels of α-tubulin is shown in lower panel. **(B)** In NCI-H1299 and PC-9 cells, the acetylation levels of α-tubulin in STMN1 overexpression and negative control groups were detected by westering blotting. Gray analysis of the acetylation levels of α-tubulin is shown in lower panel. **(C)** In NCI-H1299 and PC-9 cells, STMN1 overexpression cells were treated with DMSO or paclitaxel (10nM) for 24h, STMN1 negative control cells were treated with DMSO for 24h, the acetylation levels of α-tubulin were detected by westering blotting. Gray analysis of the acetylation levels of α-tubulin is shown in right panel.** (D)** In NCI-H1299 and PC-9 cells, STMN1 overexpression cells were treated with DMSO or paclitaxel (10nM) for 24h, STMN1 negative control cells were treated with DMSO for 24h, and transwell migration assay in these groups. The relative migrating cell numbers are analyzed in the right panel. **(E)** In NCI-H1299 and PC-9 cells, STMN1 overexpression cells were treated with DMSO or paclitaxel (10nM) for 24h, STMN1 negative control cells were treated with DMSO for 24h, and wound healing assay in these groups. The closure rates of all wounds at 48 h were analyzed and displayed in the right panel. **(F)** In NCI-H1299 and PC-9 cells, STMN1 overexpression cells were treated with DMSO or paclitaxel (10nM) for 24h, STMN1 negative control cells were treated with DMSO for 24h, the expression of EMT markers were detected by westering blotting. Gray analysis of EMT markers is shown in right panel. Abbreviations: DMSO: Dimethyl sulfoxide. *P<0.05, **P<0.01, ***P<0.001.

STMN1 is a microtubule destabilizing protein that can promote NSCLC metastasis. In this section, we explored whether STMN1 promoted NSCLC metastasis by regulating microtubule stability. As shown in Fig. 4A-4B, knockdown of STMN1 increased the acetylation levels of α-tubulin, while overexpression of STMN1 decreased the acetylation levels of α-tubulin. The results confirmed that STMN1 decreased microtubule stability. Treating the STMN1 overexpression group with paclitaxel significantly increased the expression of ace-α-tubulin, which indicated that paclitaxel could reverse the decrease in microtubule stability caused by overexpression of STMN1 (Fig. 4C). Transwell migration assays showed that treating the STMN1 overexpression group with paclitaxel could significantly reverse the increase in migrating cells caused by overexpression of STMN1 (Fig. 4D). Wound healing assays showed that treating the STMN1 overexpression group with paclitaxel could significantly reverse the enhancement of scratch healing ability (Fig. 4E). As shown in Fig. 4F, the decrease in E-cadherin and the increase in N-cadherin and vimentin caused by overexpression of STMN1 were significantly reversed after treatment with paclitaxel. These results indicated that an increase in microtubule stability significantly reversed the promotion of NSCLC cell migration by STMN1.

**Figure 5:**
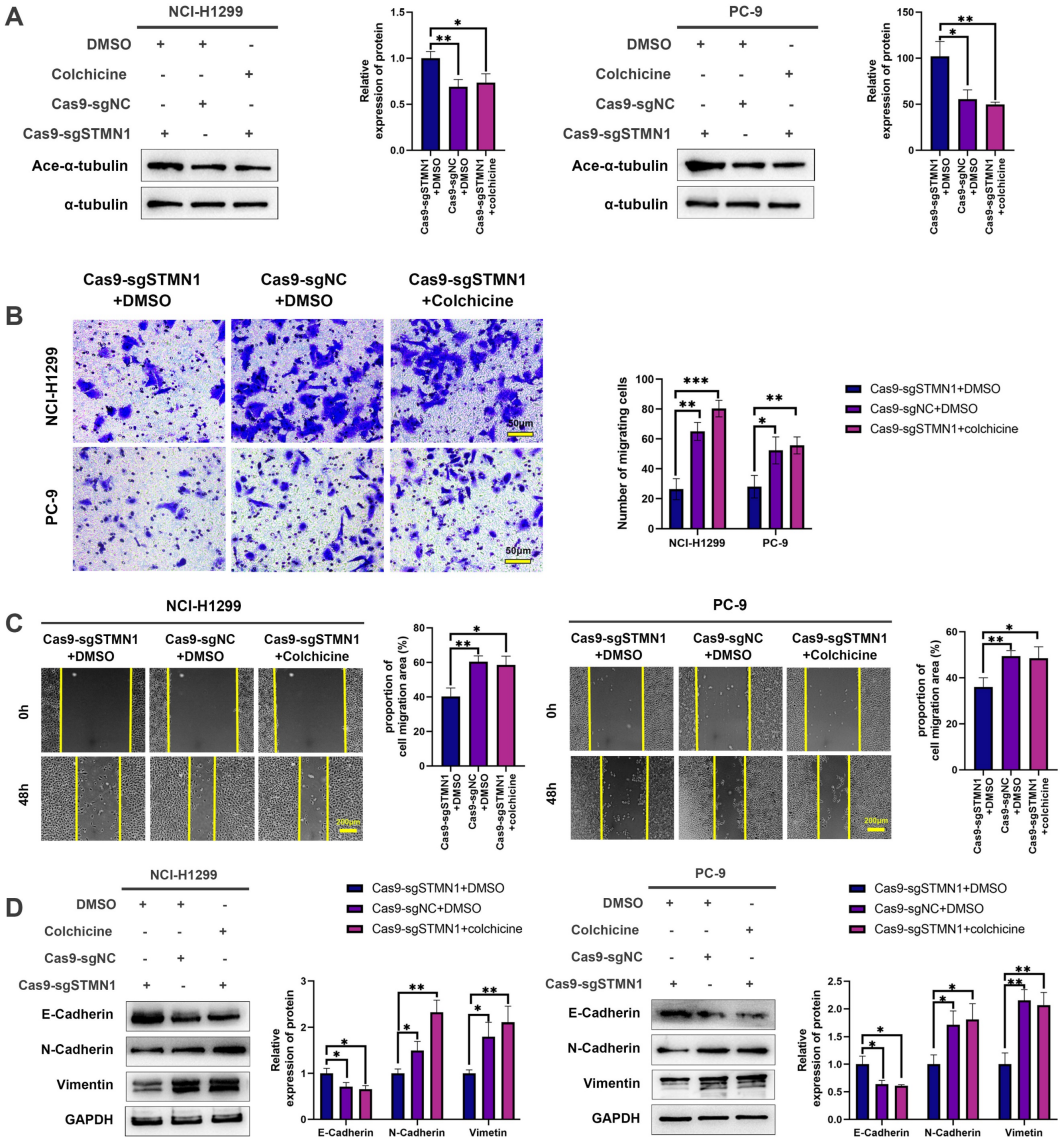
** STMN1 promotes NSCLC metastasis by regulating microtubule stability. (A)** In NCI-H1299 and PC-9 cells, STMN1 knockdown cells were treated with DMSO or colchicine (3nM) for 24h, STMN1 negative control cells were treated with DMSO for 24h, the acetylation levels of α-tubulin were detected by westering blotting. Gray analysis of the acetylation levels of α-tubulin is shown in right panel.** (B)** In NCI-H1299 and PC-9 cells, STMN1 knockdown cells were treated with DMSO or colchicine (3nM) for 24h, STMN1 negative control cells were treated with DMSO for 24h, and transwell migration assay in these groups. The relative migrating cell numbers are analyzed in the right panel.** (C)** In NCI-H1299 and PC-9 cells, STMN1 knockdown cells were treated with DMSO or colchicine (3nM) for 24h, STMN1 negative control cells were treated with DMSO for 24h, and wound healing assay in these groups. The closure rates of all wounds at 48 h were analyzed and displayed in the right panel.** (D)** In NCI-H1299 and PC-9 cells, STMN1 knockdown cells were treated with DMSO or colchicine (3nM) for 24h, STMN1 negative control cells were treated with DMSO for 24h, the expression of EMT markers were detected by westering blotting. Gray analysis of the EMT markers is shown in right panel. Abbreviations: DMSO: Dimethyl sulfoxide. *P<0.05, **P<0.01, ***P<0.001.

To further verify the above conclusion, the STMN1 knockdown group was treated with colchicine. The results showed that colchicine reversed the increase in microtubule stability caused by STMN1 knockdown (Fig. 5A). Transwell migration assays showed that treating the STMN1 knockdown group with colchicine could significantly reverse the decrease in migrating cells caused by knockdown of STMN1 (Fig. 5B). Wound healing assays showed that treating the STMN1 knockdown group with colchicine could significantly reverse the inhibition of scratch healing ability (Fig. 5C). The increase in E-cadherin and the decrease in N-cadherin and vimentin caused by knockdown of STMN1 were also significantly reversed by colchicine (Fig. 5D). Reduced microtubule stability significantly reversed the inhibitory effect of STMN1 knockdown on NSCLC cell migration. The above results indicated that STMN1 could promote NSCLC cell migration by regulating microtubule stability.

### STMN1 can interact with HMGA1

**Figure 6:**
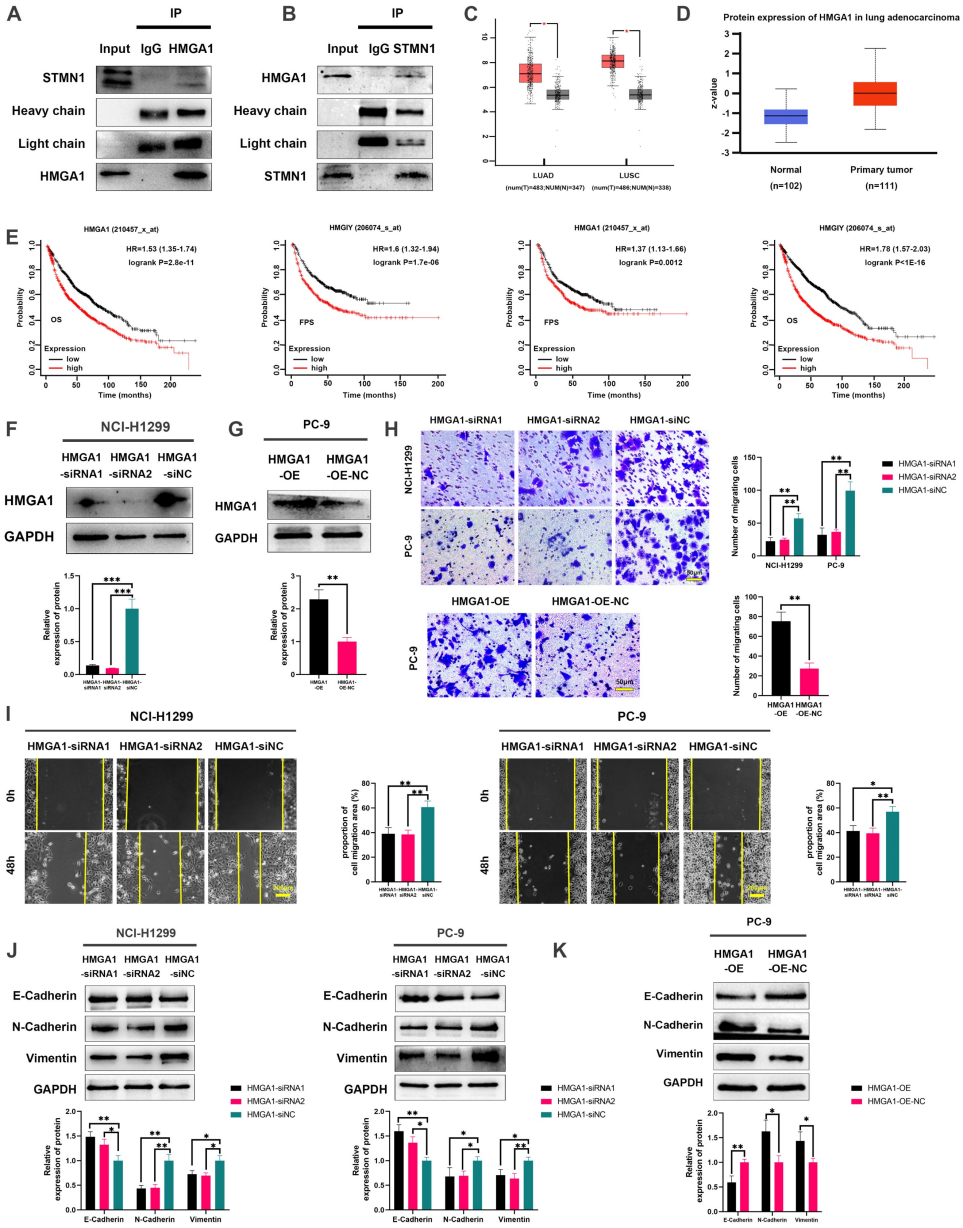
** STMN1 interacts with HMGA1 and HMGA1 promotes NSCLC metastasis. (A)** Verify the interaction between STMN1 and HMGA1 through purifying STMN1 protein combined with western blotting.** (B)** Verify the interaction between STMN1 and HMGA1 by purifying HMGA1 protein combined with western blotting. **(C)** Expression of *HMGA1* mRNA in LUAD and LUSC tumor and nontumor tissues in the GEPIA database. **(D)** Expression of HMGA1 protein in LUAD tumor and normal tissues in the CTPAC database. **(E)** The relationship between *HMGA1* mRNA and the overall survival or free progression survival of NSCLC patients in the KM plotter database. **(F)** HMGA1 knockdown efficiency in NCI-H1299 cells were tested by western blotting. Gray analysis of HMGA1 protein expression is shown in lower panel. **(G)** HMGA1 overexpression efficiency in PC-9 cells were tested by western blotting. Gray analysis of HMGA1 protein expression is shown in lower panel.** (H)** In NCI-H1299 and PC-9 cells, transwell migration assay in HMGA1 knockdown or overexpression groups and corresponding negative control groups. The relative migrating cell numbers are analyzed in the right panel.** (I)** In NCI-H1299 and PC-9 cells, wound healing assay in HMGA1 knockdown and negative control groups. The closure rates of all wounds at 48 h were analyzed and displayed in the right panel. **(J)** In NCI-H1299 and PC-9 cells, the expression of EMT markers in HMGA1 knockdown and negative control groups were detected by westering blotting. Gray analysis of EMT markers expression is shown in lower panel.** (K)** In PC-9 cells, the expression of EMT markers in HMGA1 overexpression and negative control groups were detected by westering blotting. Gray analysis of EMT markers expression is shown in lower panel. Abbreviations: LUAD: lung adenocarcinoma; LUSC: lung squamous cell carcinoma; NSCLC: non-small cell lung cancer; GEPIA: gene expression profiling interactive analysis; CTPAC: clinical proteomic tumor analysis consortium; OS: overall survival; FPS: free progression survival; KM: Kaplan Meier. EMT: epithelial-mesenchymal transition. *P<0.05, **P<0.01, ***P<0.001.

To further explore the mechanism of STMN1 in promoting NSCLC metastasis, LC‒MS/MS was used to screen the proteins interacting with STMN1. The LC‒MS/MS results illustrated that HMGA1 is a potential protein that interacts with STMN1 and is significantly associated with tumor metastasis. As shown in Fig. 6A and 6B, the relationship between STMN1 and HMGA1 was mutually verified by Co-IP combined with western blotting, and the results showed that STMN1 interacts with HMGA1.

In the GEPIA database, the result showed that *HMGA1* was highly expressed at the mRNA levels in NSCLC tissues (Fig. 6C). In the CTPAC database, the result showed that HMGA1also was highly expressed at the protein levels in NSCLC tissues (Fig. 6D). In the KM plotter database, probes 210457-x-at and 206074-s-at both illustrated that patients with high expression of HMGA1 tended to have poorer OS and FPS (Fig. 6E). To further confirm the relationship between HMGA1 and NSCLC metastasis, we successfully interfered with or overexpressed HMGA1 by siRNA and plasmid transfection, respectively (Fig. 6F and 6G). Transwell migration assays showed that interference with HMGA1 expression significantly decreased the number of migrating cells while overexpression of HMGA1 increased the number of migrating cells (Fig. 6H). Wound healing assays showed that interference with HMGA1 expression significantly inhibited scratch healing (Fig. 6I). Interference with HMGA1 expression promoted the expression of epithelial markers and decreased the expression of mesenchymal markers (Fig. 6J), while overexpression of HMGA1 caused the opposite changes (Fig. 6K). The above results indicated that STMN1 interacts with HMGA1 and that HMGA1 promotes NSCLC metastasis.

### HMGA1 can activate STMN1 to promote NSCLC metastasis

**Figure 7:**
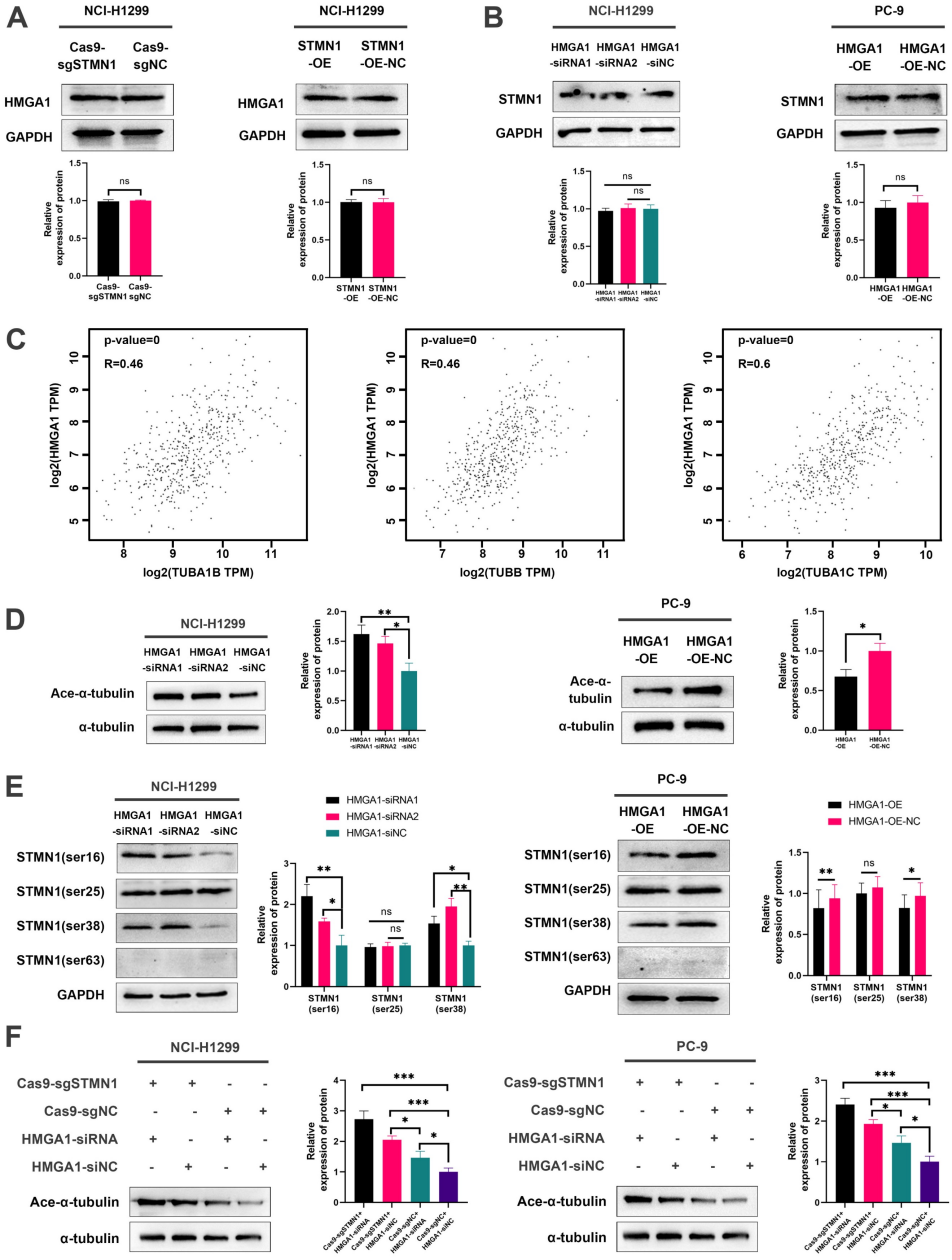
** HMGA1 can activate STMN1 to promote NSCLC metastasis. (A)** In NCI-H1299 cells, the expression of HMGA1 in STMN1 knockdown or overexpression groups and corresponding negative control groups were detected by westering blotting. Gray analysis of HMGA1 expression is shown in lower panel.** (B)** In NCI-H1299 and PC-9 cells, the expression of STMN1 in HMGA1 knockdown or overexpression groups and corresponding negative control groups were detected by westering blotting. Gray analysis of STMN1 expression is shown in lower panel.** (C)** Significant correlation genes with HMGA1 in the GEPIA database.** (D)** In NCI-H1299 and PC-9 cells, the acetylation levels of α-tubulin in HMGA1 knockdown or overexpression groups and corresponding negative control groups were detected by westering blotting. Gray analysis of the acetylation levels of α-tubulin is shown in right panel.** (E)** In NCI-H1299 and PC-9 cells, expression of STMN1 phosphorylation protein in HMGA1 knockdown or overexpression groups and corresponding negative control groups were detected by westering blotting. Gray analysis of the acetylation levels of α-tubulin is shown in right panel. **(F)** In NCI-H1299 and PC-9 cells, the acetylation levels of α-tubulin in only STMN1 knockdown group, only HMGA1 knockdown group, simultaneous STMN1 and HMGA1 knockdown group and negative group were detected by westering blotting. Gray analysis of STMN1 expression is shown in right panel. Abbreviations: GEPIA: gene expression profiling interactive analysis. *P<0.05, **P<0.01, ***P<0.001.

To evaluate the regulatory relationship between STMN1 and HMGA1, we first detected the expression of HMGA1 after knockdown or overexpression of STMN1. The results showed that knockdown or overexpression of STMN1 had no significant effects on HMGA1 (Fig. 7A). Subsequent experimental results suggested that the total protein of STMN1 also had no significant changes after interference or overexpression of HMGA1 (Fig. 7B). However, we found that HMGA1 was closely correlated with the microtubule-related genes TUBA1B, TUBB and TUBA1C in the GEPIA database, and the correlation coefficients were higher than 0.4 (Fig. 7C). The above findings suggested that HMGA1 may be related to microtubule stability, which was subsequently confirmed. Our results indicated that knockdown of HMAG1 increased microtubule stability, while overexpression of HMGA1 decreased microtubule stability (Fig. 7D).

Considering that STMN1 is a microtubule destabilizing protein, we speculated that HMGA1 regulated microtubule stability by activating STMN1 through interacting with it. The results showed that the phosphorylation levels of STMN1 on Ser16 and Ser38 were increased after knockdown of HMGA1, but the phosphorylation levels of STMN1 on Ser25 and Ser63 had no significant change (Fig. 7E). In the HMGA1 overexpression group, the phosphorylation levels of STMN1 at Ser16 and Ser38 were significantly decreased, while there was no significant change at Ser25 and Ser63 (Fig. 7F).

To further verify whether HMGA1 regulated microtubule stability by interacting with STMN1, we detected the expression of ace-α-tubulin after knockdown of only STMN1 or HMGA1 or simultaneous knockdown. The results showed that simultaneous knockdown of STMN1 and HMGA1 had the greatest impact on microtubule stability, and only knockdown of STMN1 had a greater influence on microtubule stability than knockdown of only HMGA1 (Fig. 7G). The above results indicated that HMGA1 interacts with STMN1 can decrease microtubule stability by downregulating the phosphorylation level of STMN1 at Ser16 and Ser38.

### STMN1 promotes NSCLC metastasis by activating the p38MAPK/STAT1 signaling pathway

**Figure 8:**
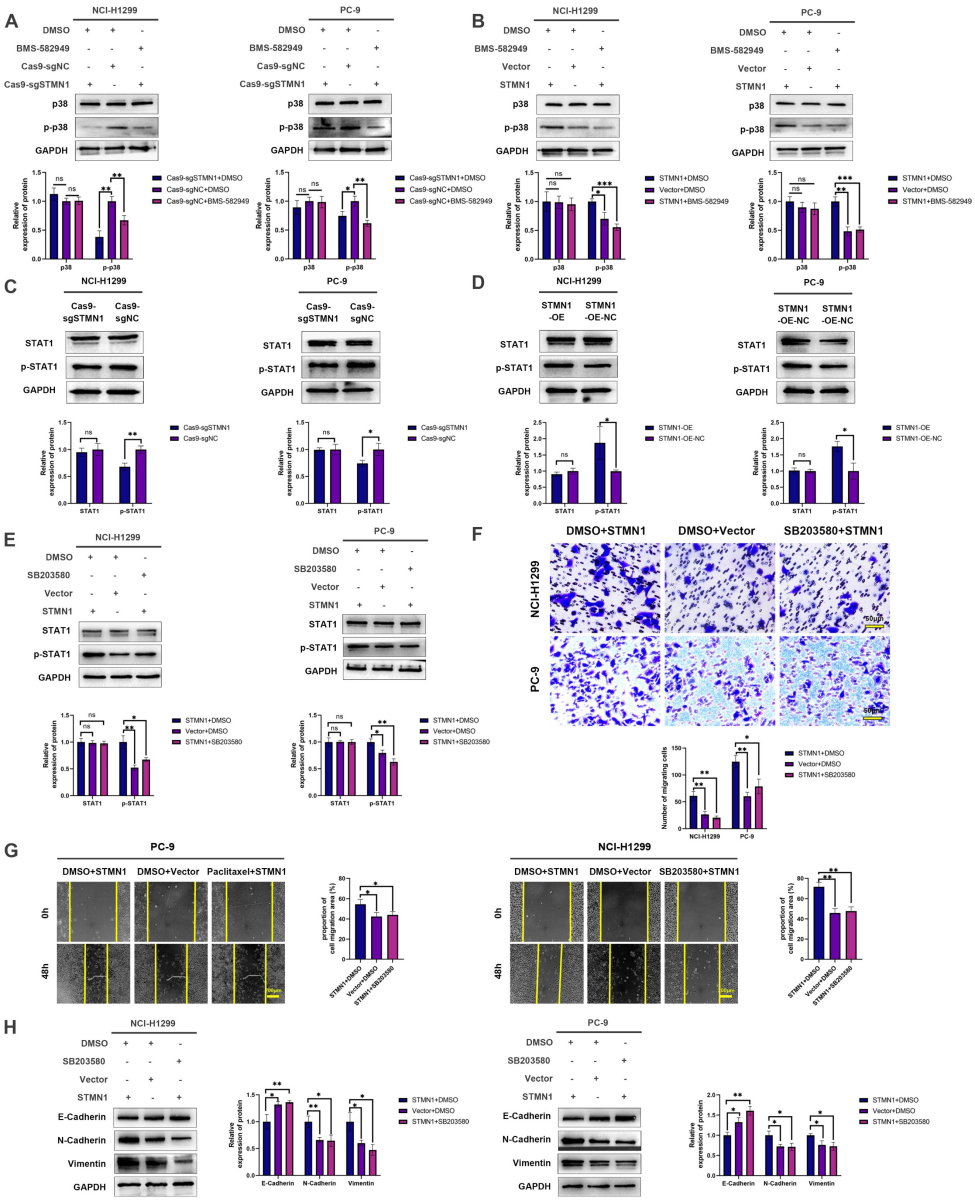
** STMN1 promotes NSCLC metastasis by activating the p38MAPK/STAT1 signaling pathway. (A)** In NCI-H1299 and PC-9 cells, STMN1 knockdown cells were treated with DMSO or BMS582949 (10 nM) for 24h, STMN1 negative control cells were treated with DMSO for 24h, the total p38 and phosphorylated p38 were detected by westering blotting. Gray analysis of the total p38 and phosphorylated p38 are shown in lower panel. **(B)** In NCI-H1299 and PC-9 cells, STMN1 overexpression cells were treated with DMSO or BMS582949 (10 nM) for 24h, STMN1 negative control cells were treated with DMSO for 24h, the total p38 and phosphorylated p38 were detected by westering blotting. Gray analysis of the total p38 and phosphorylated p38 are shown in lower panel. **(C)** In NCI-H1299 and PC-9 cells, the total STAT1 and phosphorylated STAT1 in STMN1 knockdown and negative control groups were detected by westering blotting. Gray analysis of the total STAT1 and phosphorylated STAT1 are shown in lower panel.** (D)** In NCI-H1299 and PC-9 cells, the total STAT1 and phosphorylated STAT1 in STMN1 overexpression and negative control groups were detected by westering blotting. Gray analysis of the total STAT1 and phosphorylated STAT1 are shown in lower panel. **(E)** In NCI-H1299 and PC-9 cells, STMN1 overexpression cells were treated with DMSO or SB203580 (0.5 µM) for 24h, STMN1 negative control cells were treated with DMSO for 24h, the total STAT1 and phosphorylated STAT1 were detected by westering blotting. Gray analysis of the total STAT1 and phosphorylated STAT1 are shown in lower panel. **(F)** In NCI-H1299 and PC-9 cells, STMN1 overexpression cells were treated with DMSO or SB203580 (0.5 µM) for 24h, STMN1 negative control cells were treated with DMSO for 24h, and transwell migration assay in these groups. The relative migrating cell numbers are analyzed in the lower panel. **(G)** In NCI-H1299 and PC-9 cells, STMN1 overexpression cells were treated with DMSO or SB203580 (0.5 µM) for 24h, STMN1 negative control cells were treated with DMSO for 24h, and wound healing assay in these groups. The closure rates of all wounds at 48 h were analyzed and displayed in the right panel.** (H)** In NCI-H1299 and PC-9 cells, STMN1 overexpression cells were treated with DMSO or SB203580 (0.5 µM) for 24h, STMN1 negative control cells were treated with DMSO for 24h, and the expression of EMT markers were detected by westering blotting. Gray analysis of the EMT markers is shown in right panel. Abbreviations: DMSO: Dimethyl sulfoxide**.** *P<0.05, **P<0.01, ***P<0.001.

In our previous study 29, we found that the expression of STMN1 was positively correlated with the phosphorylation level of p38MAPK. In this study, we used the p38MAPK phosphorylation inhibitor BMS-582949 as a positive control to further validate the relationship between STMN1 and p38MAPK. The results showed that the expression of STMN1 was positively related to the phosphorylation level of p38MAPK (Fig. 8A and 8B). Considering that STAT1 is a downstream molecule of p38MAPK that can be phosphorylated and activated by p38MAPK at the S727 site^40^, we speculated that STMN1 can activate the p38MAPK/STAT1 signaling pathway. As shown in Fig. 8C and 8D, knockdown of STMN1 significantly decreased the activation of p38MAPK/STAT1, while overexpression of STMN1 significantly increased the activation of p38MAPK/STAT1. These results confirmed that STMN1 can promote p38MAPK/STAT1 signaling pathway activation.

To explore the role of p38MAPK/STAT1 in the progression of STMN1 promoting NSCLC metastasis, SB203580 and fludarabine were used to inhibit the activation of p38MAPK/STAT1. As shown in Fig. 8E, treatment of the STMN1 overexpression group with SB203580 reversed the increase in STAT1 phosphorylation caused by overexpression of STMN1. Transwell migration assays indicated that the increase in migrating cells caused by overexpression of STMN1 could be significantly reversed by SB203580 (Fig. 8F). Wound healing assays indicated that the enhancement of scratch healing ability caused by overexpression of STMN1 could be significantly reversed by SB203580 (Fig. 8G). Certainly, the decrease in epithelial markers and the increase in mesenchymal markers caused by overexpression of STMN1 were also reversed by SB203580 (Fig. 8H).

**Figure 9:**
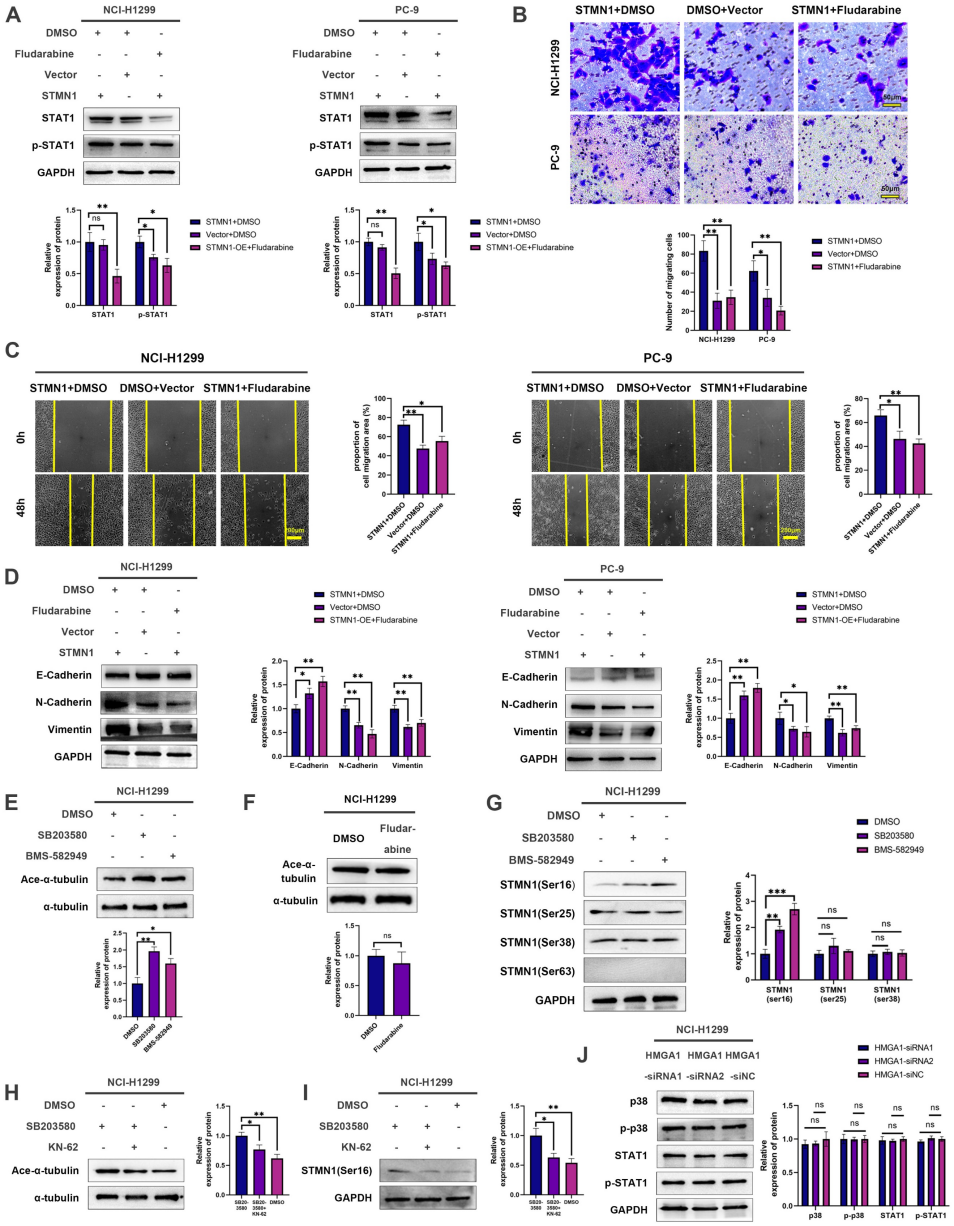
** There is a positive feedback loop between STMN1 and p38MAPK. (A)** In NCI-H1299 and PC-9 cells, STMN1 overexpression cells were treated with DMSO or fludarabine (50 µM) for 24h, STMN1 negative control cells were treated with DMSO for 24h, the total STAT1 and phosphorylated STAT1 were detected by westering blotting. Gray analysis of the total STAT1 and phosphorylated STAT1 are shown in lower panel. **(B)** In NCI-H1299 and PC-9 cells, STMN1 overexpression cells were treated with DMSO or fludarabine (50 µM) for 24h, STMN1 negative control cells were treated with DMSO for 24h, and transwell migration assay in these groups. The relative migrating cell numbers are analyzed in the lower panel. **(C)** In NCI-H1299 and PC-9 cells, STMN1 overexpression cells were treated with DMSO or fludarabine (50 µM) for 24h, STMN1 negative control cells were treated with DMSO for 24h, and wound healing assay in these groups. The closure rates of all wounds at 48 h were analyzed and displayed in the right panel. **(D)** In NCI-H1299 and PC-9 cells, STMN1 overexpression cells were treated with DMSO or fludarabine (50 µM) for 24h, STMN1 negative control cells were treated with DMSO for 24h, and the expression of EMT markers were detected by westering blotting. Gray analysis of the EMT markers is shown in right panel. **(E)** NCI-H1299 cells was treated with DMSO, SB203580 (0.5 µM) and BMS-582949 (10 nM) for 24h, respectively, and the acetylation levels of α-tubulin in each group were detected by westering blotting. Gray analysis of the acetylation levels of α-tubulin is shown in lower panel.** (F)** NCI-H1299 cells was treated with DMSO and fludarabine (50 µM) for 24h, respectively, and the acetylation levels of α-tubulin in each group were detected by westering blotting. Gray analysis of the acetylation levels of α-tubulin is shown in lower panel. **(G)** NCI-H1299 cells was treated with DMSO, SB203580 (0.5 µM) and BMS-582949 (10 nM) for 24h, respectively, and the expression of STMN1 phosphorylation protein were detected by westering blotting. Gray analysis of STMN1 phosphorylation protein is shown in right panel. **(H)** NCI-H1299 cells was treated with DMSO, SB203580 (0.5 µM) + KN62 (1 µM) and BMS-582949 (10 nM) for 24h, respectively, and the acetylation levels of α-tubulin in each group were detected by westering blotting. Gray analysis of the acetylation levels of α-tubulin is shown in right panel. **(I)** NCI-H1299 cells was treated with DMSO, SB203580 (0.5 µM) + KN62 (1 µM) and BMS-582949 (10 nM) for 24h, respectively, and the phosphorylation of STMN1 at Ser16 in each group were detected by westering blotting. Gray analysis of the phosphorylation of STMN1 at Ser16 is shown in right panel. **(J)** In NCI-H1299 cells, expression of total p38, phosphorylated p38, total STAT1 and phosphorylated STAT1 in HMGA1 knockdown and negative control group were detected by western blotting. Gray analysis of total p38, phosphorylated p38, total STAT1 and phosphorylated STAT1 is shown in right panel. Abbreviations: DMSO: Dimethyl sulfoxide**.** *P<0.05, **P<0.01, ***P<0.001.

Subsequently, the STMN1 overexpression group was treated with fludarabine, and the results showed that fludarabine could significantly reverse the activation of STAT1 caused by overexpression of STMN1 (Fig. 9A). Inhibited STAT1 activation significantly reversed the increase in migrating cells caused by overexpression of STMN1 (Fig. 9B). Wound healing assays indicated that the enhancement of scratch healing ability caused by overexpression of STMN1 could be significantly reversed by fludarabine (Fig. 9C). The decrease in E-cadherin and the increase in N-cadherin and vimentin caused by STMN1 overexpression were also reversed by fludarabine (Fig. 9D). The above results all indicated that STMN1 could promote NSCLC cell migration by activating the p38MAPK/STAT1 signaling pathway.

### There is a positive feedback loop between STMN1 and p38MAPK

As mentioned above, STMN1 can promote NSCLC metastasis by regulating microtubule stability and activating the p38MAPK/STAT1 signaling pathway. However, whether STMN1 promotes NSCLC metastasis via p38MAPK/STAT1 depends on microtubule stability needs further exploration. As shown in Fig. 9E and 9F, inhibition of the catalytic activity of p38 MAPK by SB203580 and BMS-582949 could increase microtubule stability, but there was no influence on microtubule stability after inhibition of STAT1 activity by fludarabine. This result indicated that STMN1 promoted NSCLC metastasis via the p38MAPK/STAT1 signaling pathway, which can be independent of microtubule stability.

Subsequently, the relationship between STMN1 and p38MAPK was further evaluated, and the results showed that inhibition of p38MAPK activity could promote phosphorylation of STMN1 at Ser16 (Fig. 9G). To confirm whether p38MAPK decrease microtubule stability through inhibiting the phosphorylation of STMN1 at Ser16, a calmodulin kinase inhibitor KN-62 was used to inhibit the phosphorylation of STMN1. The results showed that inhibiting the activity of calmodulin kinase could reverse the decrease in microtubule stability caused by the p38MAPK inhibitor SB203580 and the increase of phosphorylation of STMN1 at Ser16 (Fig. 9H and 9I). The results indicated that p38MAPK can decrease microtubule stability through regulating STMN1 phosphorylation, and the calmodulin kinase maybe located in the middle of the p38-STMN1 phosphorylation regulation or maybe independent of this process. The above results showed that there was a positive feedback loop among STMN1, p38MAPK and microtubules, which synergistically promoted NSCLC metastasis.

Finally, we detected the relationship between HMGA1 and the p38MAPK/STAT1 signaling pathway, but the result showed that the activation of the p38MAPK/STAT1 signaling pathway did not change after HMGA1 knockdown (Fig. 9J). We speculated that the reason for this result was the existence of other regulatory relationships between HMGA1 and the p38MAPK/STAT1 signaling pathway.

## Discussion

Our previous study showed that STMN1 was highly expressed in NSCLC tissues and positively related to lymph node metastasis. Here, we found that *STMN1* mRNA was also highly expressed in NSCLC tumor tissues. Unfortunately, the clinical information of these NSCLC patients was incomplete. As shown in Figure 1, bioinformatics analysis also showed that STMN1 is highly expressed in NSCLC and is associated with poor prognosis. The above results indicated that STMN1 was an oncogene and promoted NSCLC metastasis. However, a study published in 2012 suggested that overexpression of STMN1 inhibited cell migration^20^. In the present study, we confirmed that STMN1 promoted NSCLC metastasis through *in vivo* and *in vitro* experiments.

It is well known that STMN1 is a microtubule destabilizing protein, and its dephosphorylated form can reduce microtubule stability by promoting microtubule depolymerization, while its phosphorylated form can increase microtubule stability by inhibiting microtubule depolymerization. Microtubule dynamics are also considered closely associated with tumor metastasis. A review reported that a "stathmin-microtubule dynamics-EMT" axis may exist in the development of cancer^41^, and most studies have defaulted the biological process of “STMN1-microtubule dynamics-EMT” without conducting more detailed research. Here, we used paclitaxel and colchicine to more systematically verify that STMN1 promoted NSCLC metastasis by regulating microtubule stability.

The results of LC‒MS/MS illustrated that HMGA1 was a potential protein interacting with STMN1. HMGA1 belongs to the high mobility histone family^42^, ^43^ and is significantly associated with tumor metastasis^27^, ^28^. As shown in Figure 6, we adopted co-IP combined with western blotting to verify the interaction between STMN1 and HMGA1. Subsequent bioinformatics analysis suggested that HMGA1 was highly expressed in NSCLC tissues and was highly associated with worse prognosis. Transwell migration assays, wound healing assays and the expression of EMT markers all confirmed that HMGA1 promoted NSCLC metastasis.

Considering that HMGA1 was highly associated with tumor metastasis and that HMGA1 interacted with STMN1, we speculated that HMGA1 may be involved in the biological process by which STMN1 promotes NSCLC metastasis. As shown in Fig. 7C, HMGA1 was highly coexpressed with tubulin-related genes, which indicated that HMGA1 may be correlated with microtubule stability. We detected microtubule stability and STMN1 phosphorylation levels after knockdown or overexpression of HMGA1, and the results showed that HMGA1 could decrease microtubule stability and regulate the phosphorylation of STMN1 at Ser16 and Ser38. These results indicated that HMGA1 could decrease microtubule stability by regulating the phosphorylation level of STMN1. The results shown in Fig. 7F further confirmed that HMGA1 reduced microtubule stability by inhibiting STMN1 phosphorylation. The above results showed that the interaction between HMGA1 and STMN1 could decrease microtubule stability to promote NSCLC metastasis.

In our previous study^29^, we found a positive correlation between the expression of STMN1 and the activation of p38MAPK. STAT1 is a downstream molecule of p38MAPK, and it was considered a tumor suppressor gene in the past. However, an increasing number of recent studies have found that STAT1 is an oncogene in many cancers. A study reported that STAT1 in 12 tumors was significantly highly expressed, and the STAT1 higher group tended to have longer survival in ovarian cancer, rectal adenocarcinoma, sarcoma and skin melanoma, while the STAT1 lower group tended to have worse survival in kidney cancer, lung adenocarcinoma, and pancreatic cancer^44^. These results suggest that STAT1 plays different roles in different cancers. We found that STAT1 was highly expressed in NSCLC and was significantly associated with poor prognosis in the GEPIA database, which suggested that STAT1 was an oncogene in NSCLC. In this study, we found that STMN1 can activate the p38MAPK/STAT1 signaling pathway and that inhibiting the activity of the p38MAPK/STAT1 signaling pathway could reverse the promoting effect of STMN1 on NSCLC metastasis. The above results indicated that STMN1 can promote NSCLC metastasis by activating the p38MAPK/STAT1 signaling pathway.

Some studies^45^, ^46^ have reported that STAT3 competitively binds to the C-terminus of STMN1. STAT1 and STAT3 belong to the same family of signal transducers and activators of transcription, and their SH2 domains share up to 75% homology and are highly conserved. SH2 domains are involved in the phosphorylation of STAT proteins. Therefore, we speculated that there was an interaction between STMN1 and STAT1, but the results of Co-IP and LC‒MS/MS refuted this speculation. STAT1 is a transcription factor that can combine with DNA. We predicted the genes that could combine with STAT1 and found that ZEB1, a highly correlated gene for tumor metastasis, was a potential gene^47^. We predicted that the “STMN1-p38MAPK/STAT1-ZEB1-EMT” axis would promote NSCLC metastasis, and our research team is also preparing to further validate the “STAT1-ZEB1-EMT” section.

It was unclear whether there is a relationship between microtubule dynamics and the p38MAPK/STAT1 signaling pathway. To address this issue, we assessed microtubule stability after inhibiting the activity of p38MAPK/STAT1. The results showed that suppressing the activity of p38MAPK could enhance microtubule stability, whereas inhibiting STAT1 had no significant effect on microtubule stability. A study reported^45^ that the hypoxia-activated p38MAPK signaling pathway could cause dephosphorylation of STMN1 Ser16, which guided us to further detect the relationship between STMN1 phosphorylation and p38MAPK. The results showed that p38MAPK could decrease the phosphorylation of STMN1 at Ser16. As shown in Fig. 9H and 9I, inhibiting the activity of calmodulin kinase could reverse the increase of phosphorylation of STMN1 at Ser16 and the decrease in microtubule stability caused by p38MAPK inhibitors, which further indicated that p38MAPK decreased microtubule stability by regulating STMN1 phosphorylation. The above results demonstrated that there was a positive feedback loop among STMN1, p38MAPK and microtubules, which synergistically promoted NSCLC metastasis. In addition, Zhou^21^ et al. found that the activated p38MAPK pathway could promote microtubule depolymerization by activating microtubule-associated protein 4 (MAP4), indicating that STMN1 was only one of the multiple pathways by which p38MAPK promoted microtubule depolymerization.

According to our research results, there should be a certain correlation between HMGA1 and p38MAPK/STAT1. However, the activation of the p38MAPK/STAT1 signaling pathway did not change after HMGA1 knockdown (Fig. 9J). We speculated that there were other regulatory relationships between HMGA1 and the p38MAPK/STAT1 signaling pathway, and under the combined effect, no significant changes were detected in the activation of p38MAPK/STAT1 after HMGA1 knockdown.

## Conclusions

**Figure 10:**
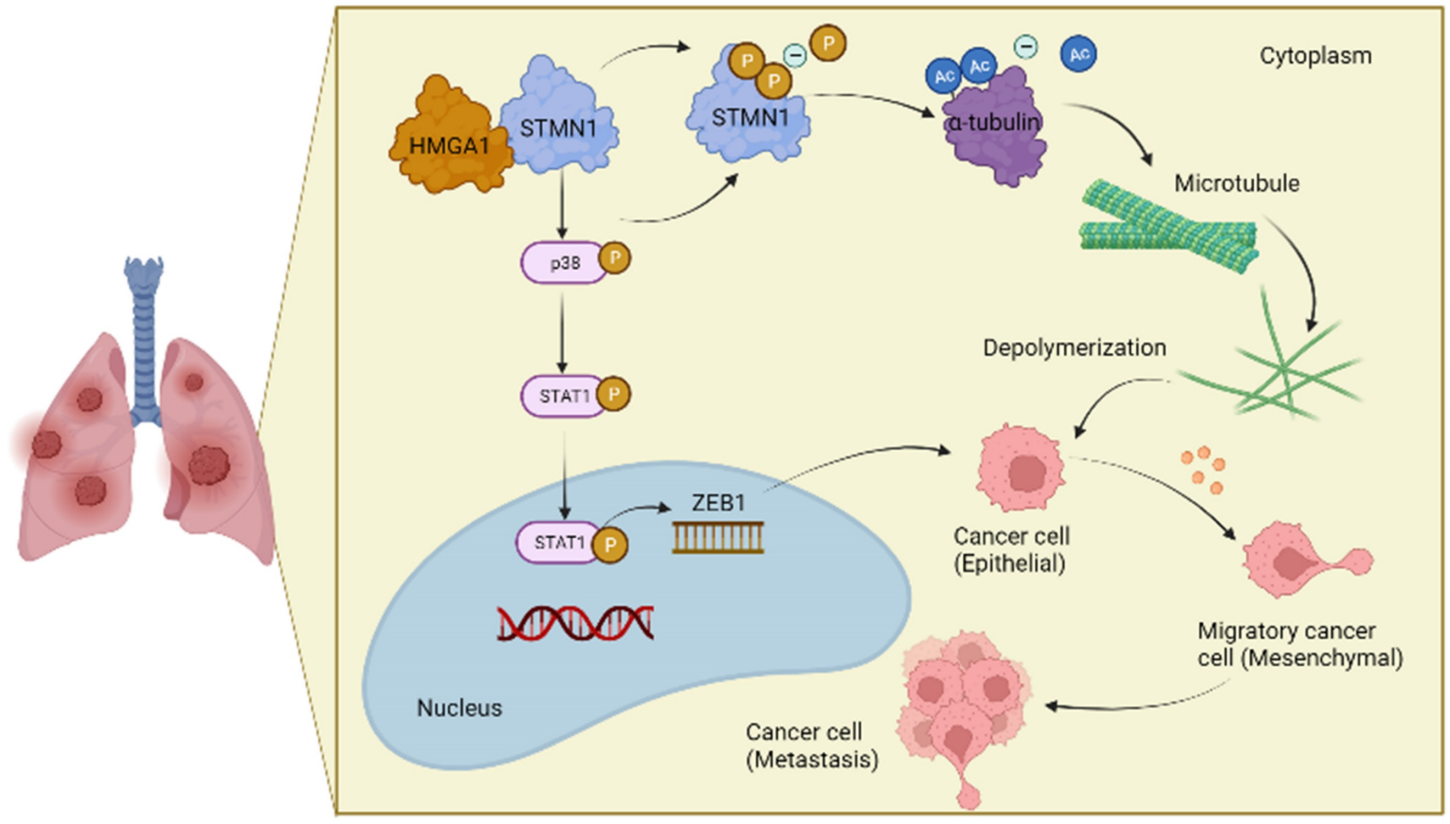
The mechanism graph of STMN1 promotes NSCLC metastasis.

In conclusion, the results obtained in this study confirmed that STMN1 promoted NSCLC metastasis and revealed that STMN1 could promote NSCLC metastasis through microtubule-dependent and nonmicrotubule-dependent mechanisms. The mechanisms by which STMN1 promoted NSCLC metastasis are shown in Fig. 10. Our conclusions provide a theoretical basis for STMN1 as a therapeutic target for inhibiting metastasis.

## Supplementary Material

Supplementary figure and tables.

## Abbreviations

Co-IP: co-immunoprecipitation
EMT: epithelial-mesenchymal transition
FBS: fetal bovine serum
HMGA1: high mobility group AT-hook 1
LUAD: lung adenocarcinoma
LUSC: lung squamous cell carcinoma
MAPK: mitogen activated protein kinases
NSCLC: non-small cell lung cancer
PAGE: polyacrylamide gel electrophoresis
PVDF: polyvinylidene fluoride
RT-qPCR: real-time quantitative polymerasechanin reaction
siRNA: small interfering RNA
STAT1: signal transducer and activator of transcription 1
GEPIA: gene expression profiling interactive analysis
CTPAC: clinical proteomic tumor analysis consortium
OS: overall survival
FPS: free progression survival
KM: Kaplan Meier
SPF: specific pathogen-free
LC‒MS/MS: liquid chromatography‒mass spectrometry/mass spectrometry
HMGA1: high mobility group AT-hook 1
DMSO: Dimethyl sulfoxide
