# Supplementary material for: STMN1 Promotes Tumor Metastasis in Non-small Cell Lung Cancer Through Microtubule-dependent And Nonmicrotubule-dependent Pathways
Source: Int J Biol Sci. 2024 Feb 7;20(4):1509–27. doi: 10.7150/ijbs.84738 (PMC10878155; doi:10.7150/ijbs.84738)
Supplement: Supplementary file 1 — Supplementary figure and tables. [file ijbsv20p1509s1.pdf]

Figure S1

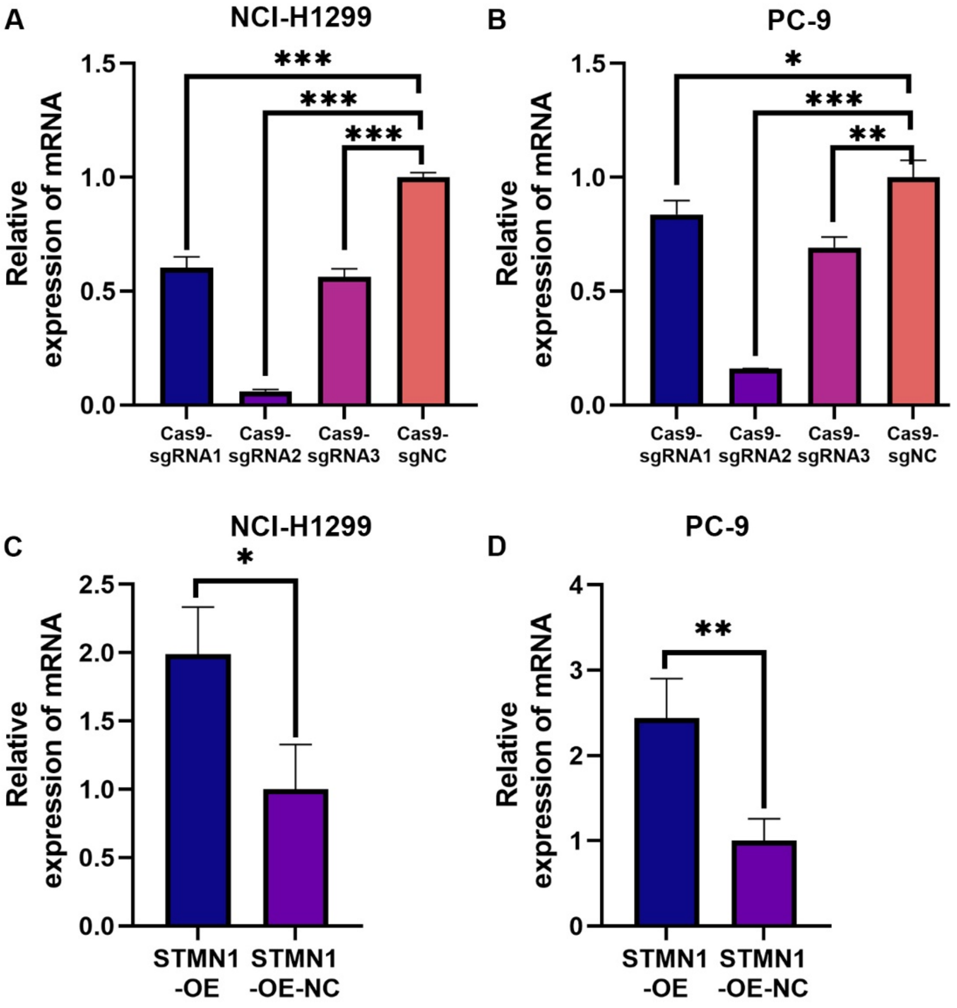

Figure S1: The efficiency of knockdown or overexpression of STMN1. (A) In NCI-H1299 cells, *STMN1* knockdown efficiency at mRNA level were tested by RT-qPCR. **(B)** In NCI-PC-9 cells, *STMN1* knockdown efficiency at mRNA level were tested by RT-qPCR. **(C)** In NCI-H1299 cells, *STMN1* overexpression efficiency at mRNA level were tested by RT-qPCR. **(D)** In NCI-PC-9 cells, *STMN1* overexpression efficiency at mRNA level were tested by RT-qPCR. Abbreviations: RT-qPCR: real-time quantitative polymerasechanin reaction. **P*<0.05, ***P*<0.01, ****P*<0.001.

Table S1. The sgRNA sequences of STMN1

sgRNA	Sequence
sgRNA1	GGAACAGATTCTTTTGACCG
SgRNA2	GAACTGGAGAAGCGTGCCTC
SgRNA3	AGCTGAGGTCTTGAAGCAGC
sgNC	CGCTTCCGCGGCCCGTTCAA

Table S2. The primer sequences of target genes

Primers	Sequence (5’to 3’)
GAPDH-F	CTCCTCCACCTTTGACGCTG
GAPDH-R	TCCTCTTGTGCTCTTGCTGG
STMN1-F	AAGAACTGGAGAAGCGTGCC
STMN1-R	GGACTTGCGTCTTTCTTCTG
HMGA1-F	CGAAGTGCCAACACCTAAGAGACC
HMGA1-R	GATGCCCTCCTCTTCCTCCTTCTC

Table S3. The siRNA sequences of HMGA1

siRNA	sequence (5'-3')
siRNA1	GAAGUGCCAACACCUAAGATT
siRNA2	CACAACUCCAGGAAGGAAATT
siNC	UUCUCCGAACGUGUCACGUTT
